# Microfluidic Isolation of Aptamers for Intracellular Measurement of Radio-Responsive Proteins

**DOI:** 10.3390/radiation5040030

**Published:** 2025-10-14

**Authors:** Xin Meng, Leah Nemzow, Yaru Han, Kechun Wen, Sally A. Amundson, Helen C. Turner, Qiao Lin

**Affiliations:** 1 Department of Mechanical Engineering, Columbia University, New York, NY 10027, USA; 2 Center for Radiological Research, Columbia University Irving Medical Center, New York, NY 10032, USA

**Keywords:** radiation biodosimetry, intracellular proteins, radiation-responsive biomarkers, microfluidic aptamer isolation

## Abstract

In large-scale radiological events, there is a need to triage affected individuals based on their biological absorbed dose. Biodosimetry measures biological responses in relation to the received dose. Radiation-responsive protein biomarkers in peripheral blood lymphocytes, especially intracellular proteins, have been validated for biodosimetry with immunochemical-based measurement methods. However, these antibody-based assays can suffer from stability and batch-to-batch variations. Aptamers are single-stranded oligonucleotide alternatives to antibodies that are stable and much smaller in size, making them ideal probes for intracellular targets. However, few aptamers have been developed against intracellular targets, and these efforts are especially hampered due to the time-consuming nature of the conventional aptamer selection method. An efficient method for isolating aptamers against intracellular radiation-responsive proteins is not available yet. Herein, we used a microfluidic aptamer isolation method to develop an aptamer against the intracellular radiation biomarker BAX in blood lymphocytes. The isolated aptamer has a dissociation constant of 6.95 nM against human BAX protein and a bright detail similarity score of 1.9 when colocalizing with anti-BAX aptamer intracellularly. The in situ labeling of the intracellular BAX protein also shows the aptamer can be used to differentiate 2.5 Gy or 3 Gy of radiation in ex vivo human and in vivo mouse peripheral blood samples exposed to X-rays. In conclusion, this proof-of-concept study indicates that the microfluidic-enabled aptamer isolation method could be used for the development of a panel of targeted intracellular proteins for radiation biodosimetry applications.

## Simple Summary

Radiation incidents, such as nuclear plant failures or terrorist attacks, can expose many people to harmful doses of radiation. To guide medical care quickly, doctors need ways to measure how much radiation a person has received. Current laboratory tests often take several days and are too slow for large-scale emergencies. In this study, we developed a new type of probe called an aptamer, which is a short piece of DNA that can fold into a shape that sticks to a specific protein inside blood cells. We focused on a protein called BAX, which increases when cells are damaged by radiation. Using a miniature device that combines all steps of the selection process on a single chip, we were able to isolate a working aptamer against BAX in less than a day, compared with weeks using traditional methods. We showed that this aptamer can recognize BAX inside human and mouse blood cells and can distinguish samples that received different radiation doses. These results demonstrate a faster and more reliable way to develop specific aptamer probes for measuring changes inside cells. In the future, this approach could help doctors rapidly identify people who need urgent treatment after radiation exposure.

## 1. Introduction

Following a mass-casualty radiological/nuclear (R/N) incident such as a nuclear power plant accident or detonation of an improvised nuclear device, rapid clinical triage of potentially exposed victims for absorbed radiation dose will be essential to help determine appropriate diagnostic and therapeutic interventions. Quick and accurate dose reconstruction within the first week will be important for early triage decisions [1].

Biological dosimetry, or biodosimetry, is the estimation of absorbed radiation dose based on measurements of observable biological changes that occur inside the body, which are reflected in biomarkers in accessible biofluids (i.e., peripheral blood, urine, saliva). Measuring these changes can achieve personalized dosage quantification and anticipate potential consequences, assisting in the planning of suitable medical therapy [2]. To date, several biodosimetry methods have been developed [3], including the dicentric chromosome assay (DCA) [4–6], histone 2AX (H2AX) [7,8], micronuclei [9,10], microRNA [11,12], lncRNA [13,14], and gene expression assays [15]. Among these methods, the DCA is regarded as the “gold standard” reference method for cytogenetic biodosimetry. However, this cell-culture-based assay is time-consuming and laborious, with a long turnaround time of 72–96 h for dose estimation, and is therefore not amenable to timely measurement of radiation exposure in a large-scale emergency [16]. Therefore, there is an important need for the development of rapid, high-throughput biodosimetry assays with high radiosensitivity and selectivity for dose assessment and triage after a large-scale radiological event [16–18].

Previously, we have used commercial antibodies to develop an imaging flow cytometry-based protein bioassay [19] that rapidly detects radio-responsive intracellular proteins in peripheral blood lymphocytes for estimation of the biological absorbed dose after radiation exposure [20]. Aptamers, single-stranded nucleic acids with high affinity and specificity to biological targets, provide an alternative category of affinity-binding probes and have been extensively developed for molecular diagnostics, therapeutics, and other applications [21,22]. Compared with antibodies, aptamers have advantageous attributes such as reversible target-binding [23], longer shelf life [24], minimal batch-to-batch variations [25], low immunogenicity [26], and ease of conjugation with functional groups [27]. Moreover, aptamers are much smaller in size, which may be important when evaluating intracellular targets, as they have better intracellular delivery efficiency [28]. However, aptamer-based methods for protein biodosimetry have seldom been explored. While aptamers have recently been used as a screening tool for identifying new biomarkers in studies by Sproull et al. [29], they have not been used directly as a measurement tool for biodosimetry, and development of aptamer-based measurement methods could provide a powerful approach for rapid biodosimetry.

We developed an efficient and cost-effective microfluidic platform to develop aptamers for intracellular protein targets, thereby achieving rapid and efficient aptamer selection via microfluidic device. The traditional aptamer selection process, systematic evolution of ligands by exponential enrichment (SELEX), is inherently time-consuming and labor-intensive, but its efficiency can be greatly enhanced through the incorporation of novel microfluidic techniques. Microfluidic SELEX offers distinct advantages over conventional bead-based approaches, including markedly shorter cycle times, reduced reagent consumption, and improved automation. Notably, fully integrated microfluidic platforms are capable of completing multiple selection rounds within a single day, whereas conventional methods often require weeks of labor-intensive processing. These advantages underscore the potential of microfluidic platforms as an efficient and scalable strategy for aptamer discovery [30–33]. In this work, we use bead-based affinity selection and amplification procedures [33] in an integrated manner to enhance the isolation efficiency, which can speed up the aptamer isolation time from 2 weeks to 1 day to facilitate the development of aptamers for radiation-responsive biomarkers of interest. This method allows for efficient and robust development of aptamers and is applicable to a broad range of intracellular targets.

As the development of aptamers for intracellular proteins remains at an early stage, this study contributes to the preliminary exploration of their potential in biodosimetry applications [34]. Aptamers that target intracellular proteins are still scarce, possibly because intracellular targets are, in general, difficult to access due to their localization. Previously developed aptamers against intracellular proteins are usually used as intramers (expressed inside the target cells using molecular biology techniques), and thus are not suitable for intracellular measurement of radiation-responsive proteins for triage purposes. Consequently, the engineering of aptamers tailored to recognize intracellular proteins emerges as a novel endeavor in advancing the field of intracellular biodosimetry.

We describe an early study toward the development of aptamers for radiation biodosimetry. We chose BAX protein as the first candidate intracellular target, as this protein has shown promising radiation response in previous biodosimetry studies [20,35,36], and this work represents one of the earliest efforts to develop aptamers against intracellular radiation-responsive proteins for biodosimetry. BAX protein or Bcl-2-associated X protein is part of the Bcl-2 (B-cell lymphoma 2) protein family that regulates cell death through apoptosis [37–39]. Here, we report the development of an aptamer for the BAX protein obtained from a bead-based microfluidic SELEX method. In this study, we focused aptamer selection on the BAX BH3 domain, a functional epitope essential for Bax–Bcl-2 family interactions. Targeting a defined epitope reduces the conformational heterogeneity associated with full-length BAX and improves the likelihood of isolating binders with consistent recognition in cells [40,41]. We have therefore used the BH3 peptide for selection while evaluating recognition of full-length BAX in cellular assays. We show that the isolated aptamer was able to bind with BAX protein with high affinity in vitro. The aptamer was also tested in vivo by examining the colocalization of the isolated aptamer with commercial antibody to BAX protein, and functionally validating the aptamer’s ability to detect radiation-induced increases in BAX protein in human and mouse blood models.

This study serves as a proof-of-principle to validate the efficacy of our advanced microfluidic SELEX methodology for the development of an intracellular aptamer that could potentially be used for large-scale, rapid biodosimetry and highlights the suitability of our microfluidic SELEX method for the complex demands of intracellular measurements. This study also demonstrates the utility of the BAX aptamer as an innovative biosensor and affirms the methodological advancements in the field of aptamer development, thus making a significant step towards broader translational applications.

## 2. Results and Discussion

### 2.1. Microfluidic Selection of Aptamers

The DNA aptamer of BAX was selected from a library consisting of a 40-nucleotide random region that is flanked by a primer region on both 5′ and 3′ ends. Instead of using the whole BAX protein, a segment of the BAX peptide was used. This 20-amino acid peptide is the BAX BH3 domain corresponding to the residues 55 to 74 of the whole protein. The BAX BH3 peptide (STKKLSECLKRIGDELDSNM) adopts a helical structure [42] and can induce apoptosis in a variety of cell line models [42]. By choosing BAX BH3 as the SELEX target, we aimed to obtain aptamers that not only bind with BAX proteins but that could also potentially be used to regulate apoptosis in future studies. Furthermore, it was believed that using a peptide instead of the whole protein would increase the specificity of the isolated aptamer, as previously reported in the literature for HIV Rev protein and Human RB protein [28,43].

**Figure 1.**
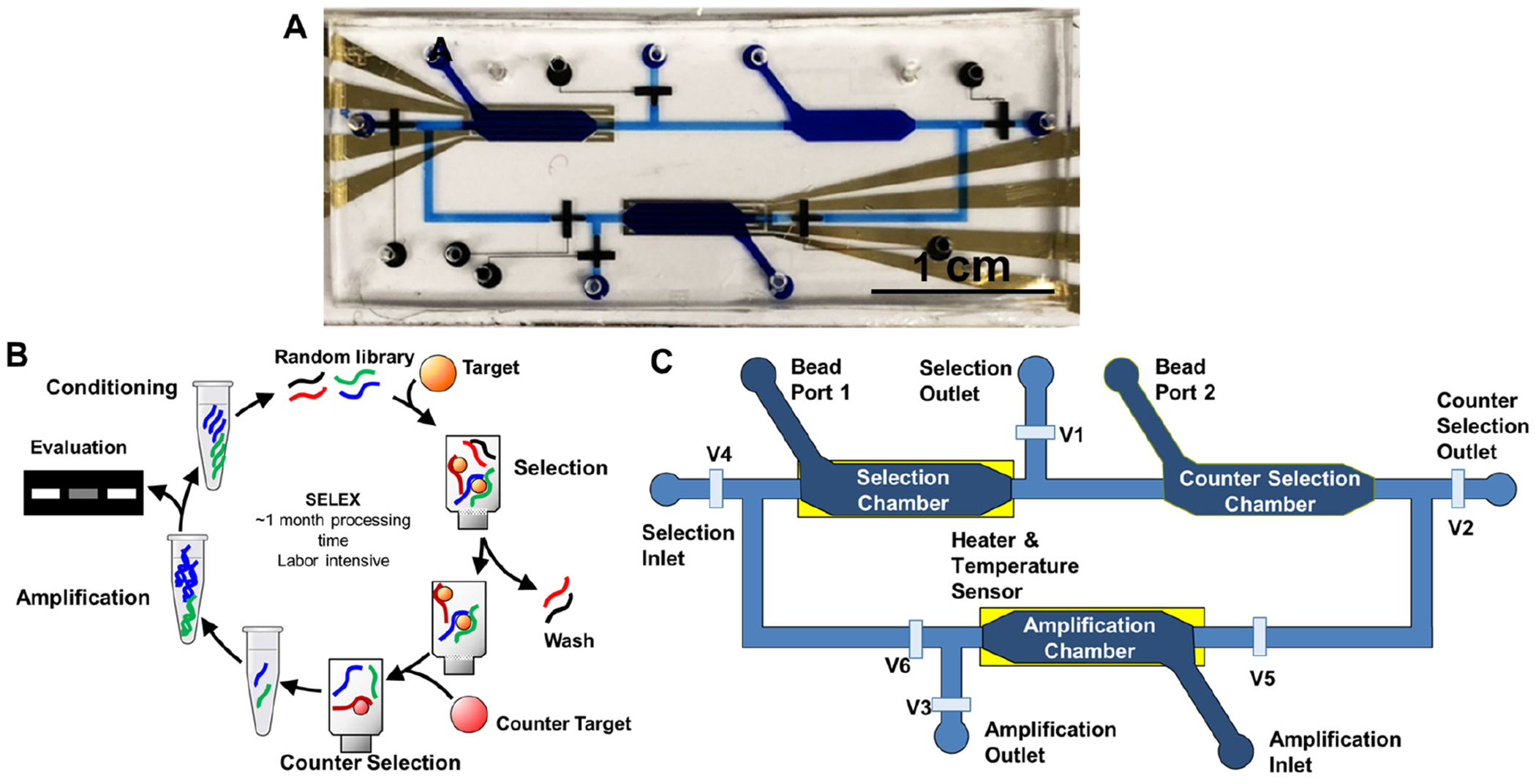
Microfluidic SELEX Design. (**A**) Picture of full-chip SELEX device. (**B**) SELEX process overview. Random library of oligonucleotides will go through selection, optional counter selection, amplification, and conditioning to yield the enriched library for the following round. This iterative process will continue for 15–20 rounds for conventional SELEX that in total will take about a month to process. (**C**) Schematics of full-chip SELEX device. V1–V6 indicate valves for flow control.

Microfluidic full-chip SELEX (Figure 1) was performed for nine rounds. The enriched pools from round 6 and round 9 were sent for next-generation sequencing (NGS). The NGS results were analyzed using Aptasuite [44] and clustered. The top nine clusters are shown in Table 1. The rest of the sequences did not have enough cluster size to be considered significant and were thus omitted.

As observed in Table 1, there are mainly three unique sequences highly enriched in both round 6 and round 9. This means that the pool is highly homogeneous and that it is unlikely that other unique sequences in the pool will be enriched. Another enrichment pattern can also be observed from this NGS result: While the red sequence (Apt2) and the green sequence (Apt3) are predominant in the respective round 6 pool and round 9 pool, the blue sequence (Apt1) is constantly present in both pools. These three sequences were considered leads and subsequently synthesized and used for the characterization. For the following experiments, full-length sequences were used, including the forward and reverse primer regions.

**Figure 2.**
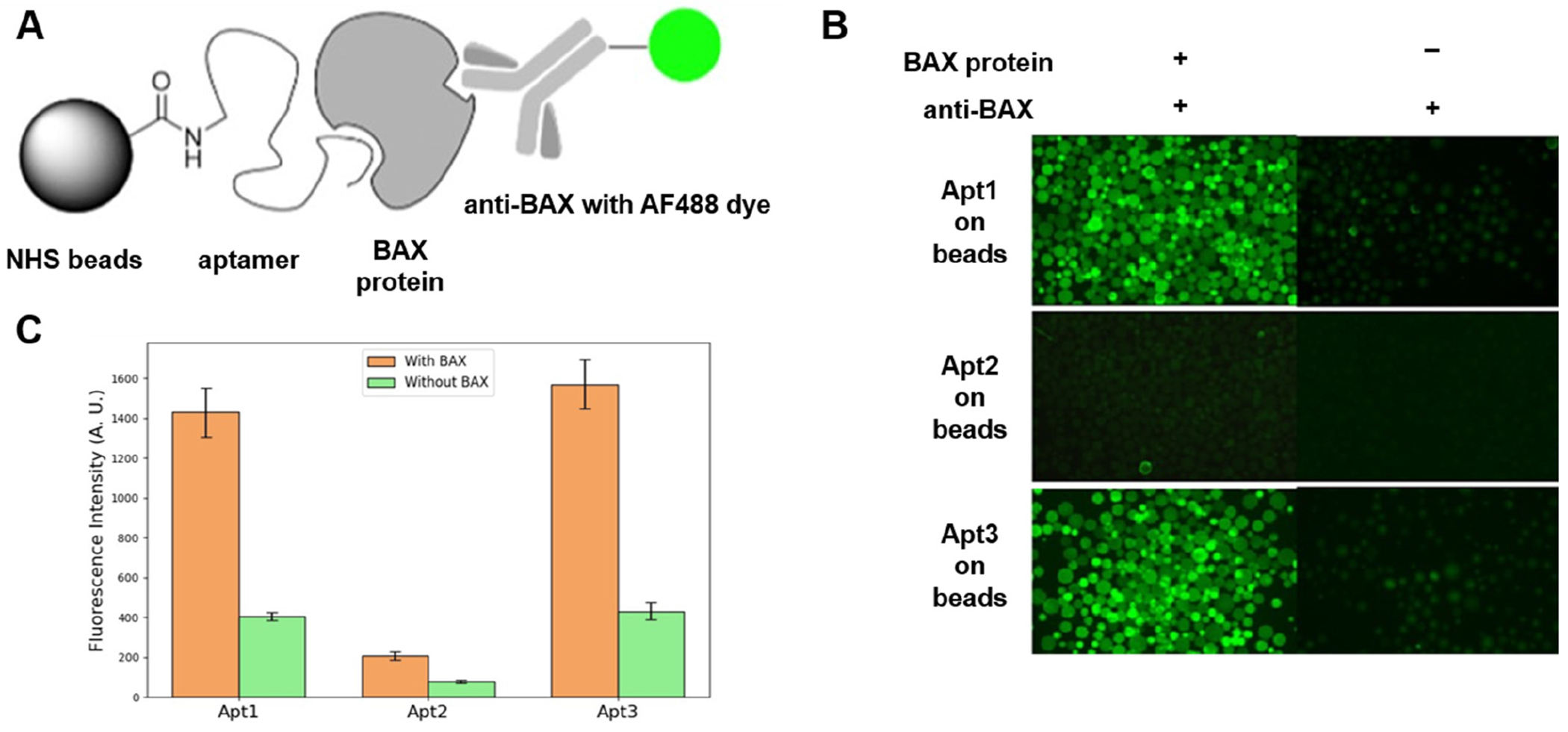
Sandwich assay. (**A**) Assay design. (**B**) Fluorescent images of bead-immobilized aptamer candidates exposed to BAX protein and anti-BAX antibody. (**C**) Average fluorescence intensity of 20 beads (error bars represent standard errors from three independent measurements).

A bead-based sandwich assay was used to characterize the in vitro binding affinity of candidate BAX proteins. Since the aptamers will ultimately be used for intracellular binding with the whole BAX proteins, we performed a bead-based sandwich assay to examine the binding capabilities of these unique sequences to the whole BAX proteins. In this assay, aptamer candidates were immobilized on the surface of agarose beads and subsequently incubated with BAX protein and a fluorescent BAX antibody (Figure 2A). This BAX antibody bonded with the residuals 3 to 16 of human BAX protein [45], which differs from the BAX BH3 sequences that the aptamer candidates were designed to bind. This eliminated the possibility of competitive binding between anti-BAX antibodies and aptamer candidates against whole BAX protein. If an aptamer candidate bonded with BAX protein, the sequential incubation would immobilize both BAX protein and the fluorescent anti-BAX on the surface of the beads, resulting in increased fluorescence.

Fluorescence images (Figure 2B) were acquired to evaluate the binding affinity of three aptamer candidates (Apt1, Apt2, and Apt3). Each bead type was subjected to two experimental conditions: (1) incubation with both BAX human recombinant protein and Alexa Fluor 488-conjugated anti-BAX mouse monoclonal antibody used as a reporter to show successful capture of BAX protein by the aptamer-coated beads (left column), and (2) incubation with the anti-BAX antibody alone in the absence of BAX protein (right column). It was observed that beads coated with Apt1 or Apt3 exhibited a strong fluorescence signal following sequential incubation with BAX protein and the anti-BAX antibody, indicating that Apt1 and Apt3 effectively recognized the BAX target. Minimal fluorescence was observed in the absence of BAX protein, suggesting negligible nonspecific interaction between either aptamer candidate or the anti-BAX antibody. In contrast, beads coated with Apt2 displayed minimal fluorescence under both conditions, indicating a lack of binding to BAX protein and no nonspecific interaction with the anti-BAX antibody. Based on these results, Apt2 was excluded from further consideration due to insufficient target affinity. Thus, we conclude that Apt1 and Apt3 are BAX-binding aptamers with minimal nonspecific interactions, with Apt3 exhibiting moderately stronger binding affinity than Apt1, but the difference was not statistically significant. The observed fluorescence intensities are attributable to specific recognition of BAX protein rather than direct interaction with the anti-BAX antibody.

Quantification of the fluorescence in these images further supports the hypothesis of minimum nonspecific interaction (Figure 2C). Inspection [46] of the primary nucleic acid sequences of Apt1 and Apt3 found that these sequences are rich in guanine, suggesting they might potentially adopt G-quadruplex structures. Thus, they are capable of forming four-stranded structures called G-quadruplexes, stabilized by Hoogsteen hydrogen bonding between a tetrad of guanine bases. After the initial sandwich assay screening, the blue (Apt1) and green (Apt3) sequences were carried over in the following experiments.

**Figure 3.**
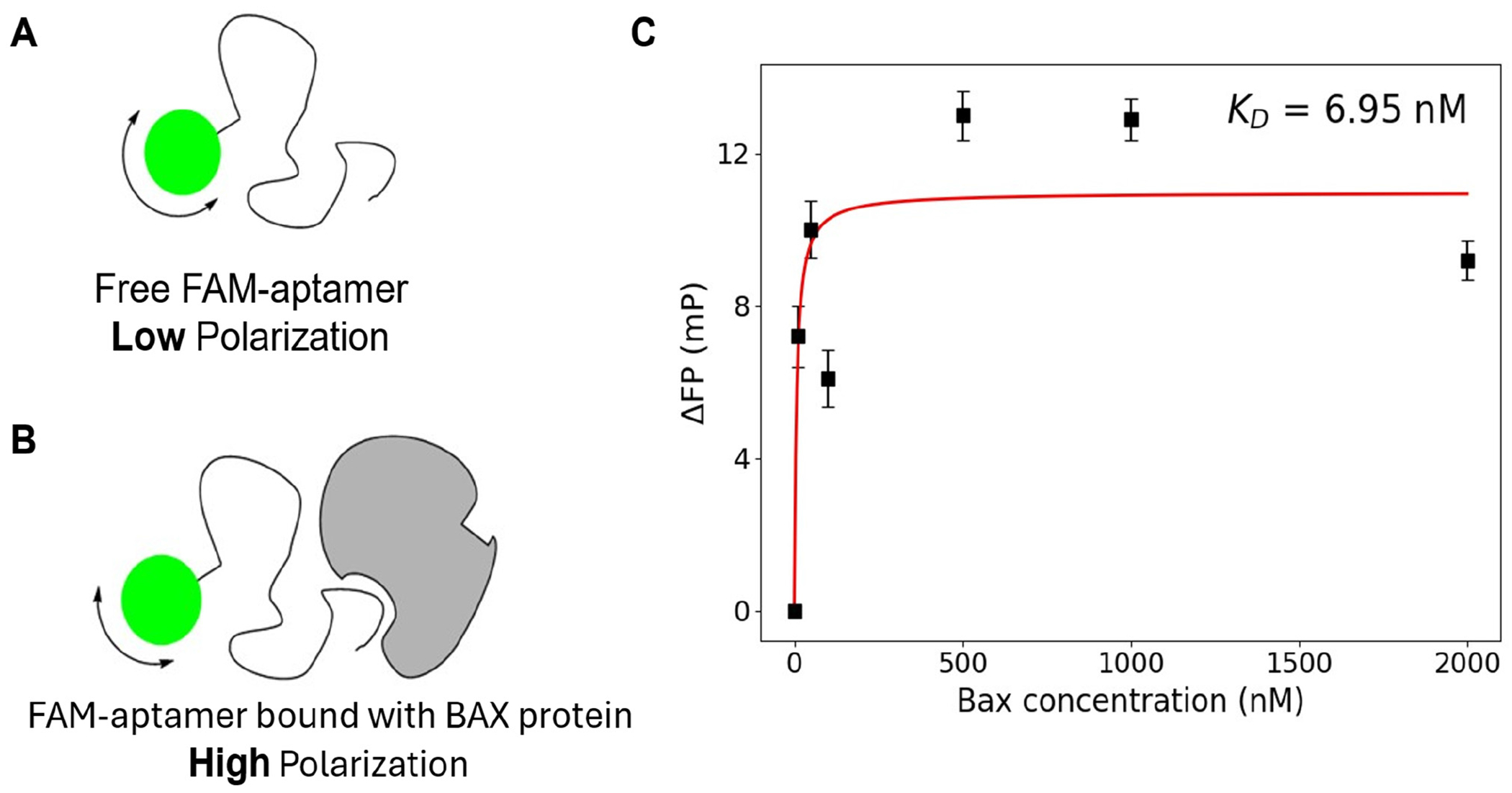
Fluorescence Polarization Assay. The design and principle of the assay are shown in (**A**) for free fluorescent aptamers and (**B**) protein target-bound fluorescent aptamer. (**C**) Titration curve of Apt3 for *K*_D_ quantification (error bars represent standard errors from three independent measurements).

To demonstrate quantitatively on a molecular level that the aptamer does indeed actually bind to the BAX protein peptide, the dissociation constant *K*_D_ was determined via a fluorescence polarization (FP) assay. The basic principle of the FP assay is that when a fluorescently labeled molecule is excited by polarized light, it emits light with a degree of polarization that is inversely proportional to the rate of molecular rotation. When a nucleic acid ligand with a fluorescent label attached (Figure 3A) is excited by polarized light at the excitation wavelength of the fluorophore, the ligand reorients significantly due to molecular tumbling during the excited state lifetime of the fluorophore. This causes the emitted light to be largely depolarized. If the ligand is bound to a protein (Figure 3B), the resulting complex tumbles much slower, and the emitted light retains its polarization. This property of fluorescence can be used to measure the interaction of a small labeled ligand (aptamer candidates in this case) with a larger protein (BAX protein in this case) [47]. The dissociation constant *K*_D_ of Apt3 was determined to be 6.95 nM (Figure 3C), while Apt1 did not produce a clear titration curve in the FP assay, likely due to assay-related interference. Given that Apt3 exhibited strong and consistent binding with a well-defined *K*_D_ curve, Apt3 was chosen for the following cell studies.

### 2.2. In Vivo Colocalization of Anti-BAX Aptamer and Antibody

**Figure 4.**
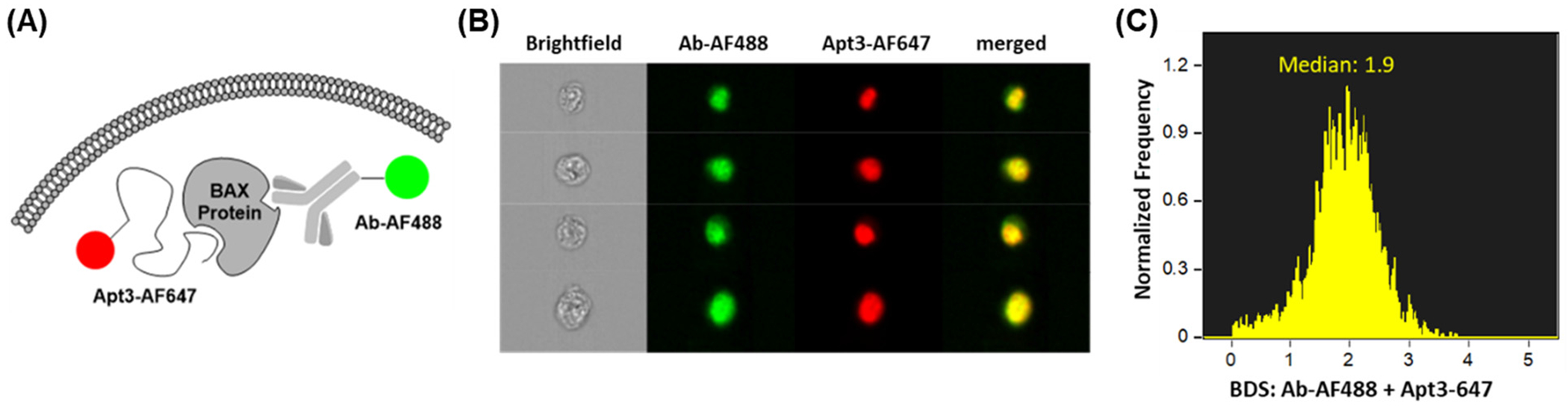
Colocalization of anti-BAX antibody and aptamer. (**A**) Design and principle of the colocalization assay: commercial antibody (Ab-AF488) and aptamer (Apt3-AF647) are designed to target different amino acid regions of intracellular BAX protein. (**B**) Representative gallery of doublepositive TK6 cells that were fixed and co-labeled with Ab-AF488 and Apt3-AF647 and acquired by imaging flow cytometry. (**C**) Bright Detail Similarity score of co-labeled double-positive TK6 cells.

To verify whether Apt3 successfully binds to BAX protein in an in vivo, cellular context for intracellular measurement, we performed a colocalization study. Because the SELEX target was the BAX BH3 domain, a key epitope for Bax–Bcl-2 interactions, it was important to confirm recognition of full-length BAX in cells. As shown in Figure 4A, fixed human lymphoblastoid TK6 cells were co-labeled with both, Apt3 conjugated to AF647, and a validated commercial antibody conjugated to AF488, each designed to target different BAX protein sites (amino acids 55–74 and 3–16, respectively). The co-labeled cells were subsequently interrogated by imaging flow cytometry. Figure 4B shows that the Ab-AF488 signal and Apt3-AF647 signal share localization patterns within the co-labeled TK6 cells. Aptamer and antibody colocalization were quantified by bright detail similarity (BDS), a metric of correlation between the localized bright spots (see Materials and Methods). Figure 4C shows that the median BDS score value of double-positive cells is 1.9, a value appreciably over 0, indicating relatively successful colocalization of Apt3-647 and Ab-AF488 in the TK6 cell line.

### 2.3. Functional Validation of Intracellular BAX Labeling in Human and Mouse Peripheral Blood

**Figure 5.**
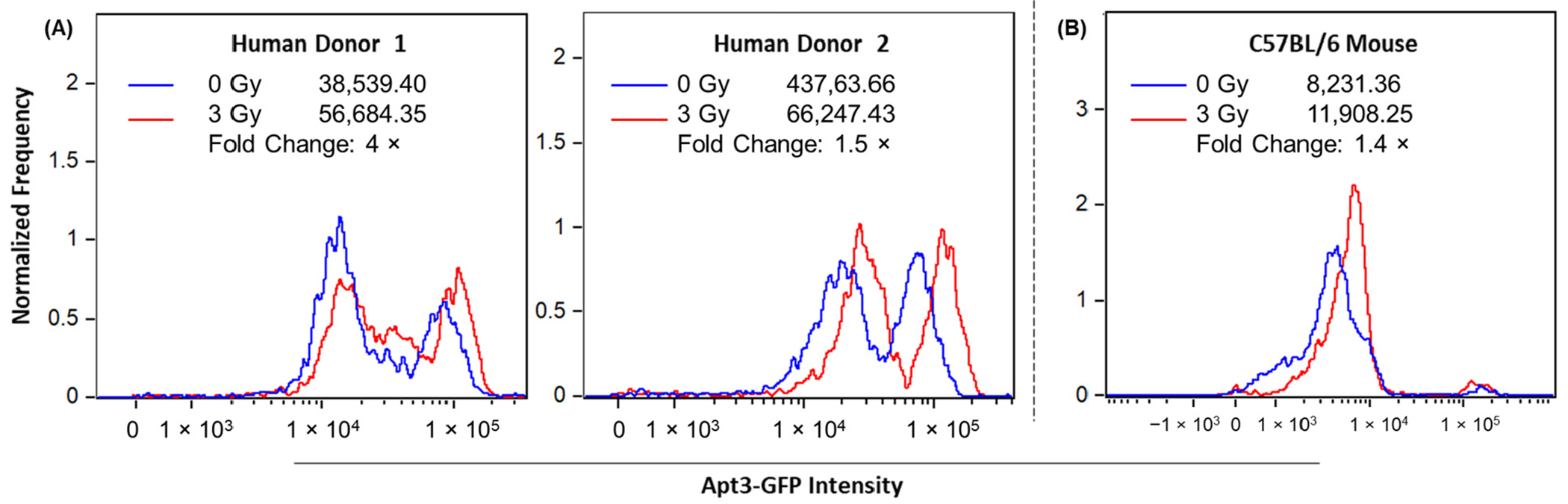
Apt3 detection of intracellular BAX protein in human ex vivo and mouse in vivo peripheral blood models. (**A**) Whole blood from two human donors was X-irradiated at 0 and 3 Gy and cultured for 24 h. (**B**) C57BL/6 mice were X-irradiated with 0 and 2.5 Gy (one mouse at each dose) and sacrificed after 24 h. MFI of Apt3-GFP in peripheral blood leukocytes at each dose was measured by imaging flow cytometry. In the representative histograms of Apt-GFP signal intensity, peaks correspond generally to leukocyte populations of varying sizes and the amount of generated fluorescence. In human leukocytes, the peak on the left generally represents lymphocytes, and the peak on the right represents granulocytes. Within mouse leukocytes, granulocytes are infrequent, and the large peak generally represents only lymphocytes. Fold changes of dose-dependent Apt3-GFP signal in human and mouse leukocytes were calculated as (3 Gy/0 Gy) or (2.5 Gy/0 Gy), respectively.

To validate Apt3’s ability to detect biologically functional responses of intracellular BAX protein in a cellular model, we examined radiation-induced responses of Apt3-labelled BAX protein in human and mouse peripheral blood leukocytes 24 h after X-irradiation. These human ex vivo and mouse in vivo models for testing the radiation response of BAX were previously validated using commercial antibodies [35] (Figure S1). Figure 5A shows that in Apt3-labelled leukocytes from two human donors, mean fluorescence intensity (MFI) values of the irradiated samples (3 Gy, shown in red) are higher on the intensity scale than those of the mock-irradiated samples (0 Gy, shown in blue), increasing to ~1.5-fold. Figure 5B shows a similar result in an in vivo C57BL/6 mouse model, where the MFI has increased 1.4× after 2.5 Gy X-irradiation. These findings suggest that Apt3 may act as a molecular probe to detect biological upregulation of BAX protein and that this anti-human BAX aptamer may cross-react with BAX protein in other species with conserved homology, suggesting that these early data warrant further studies to extensively optimize staining and interrogation protocols for biomarker validation in human and animal models for applications in point-of-care intracellular biodosimetry on a microfluidic platform.

## 3. Experimental Section

### 3.1. Aptamer Selection and In Vitro Characterizations

#### Fabrication and packaging of microfluidic SELEX chips.

The full-chip SELEX chip mold was fabricated using the same methods described previously [33]. Briefly, the mold was fabricated with photolithography using SU8 photoresists, and the fluidic channel layer and the control valve layer were then made with Polydimethylsiloxane (PDMS) using the molds. Heaters and sensors were patterned on glass slides with photolithography and “lift-off” metal etching. The whole chip was assembled with the oxygen plasma bonding method.

#### Microfluidic SELEX setup.

The full-chip SELEX system consists of an integrated chip, a syringe pump, a nitrogen tank, a multimeter, and a power supply. The full-chip SELEX chip (Figure 1A) is the centerpiece, while the syringe pump supplies the fluid source. The nitrogen tank is used together with a splitter to actuate the valves on the full-chip SELEX chip with pneumatic control. The multimeter and power supply are controlled by LabView codes for temperature manipulations in the chambers of the full-chip SELEX chip. The chip integrates three bead-packed chambers for selection, counter-selection, and amplification, connected by 20 µm high microchannels that confine agarose beads (~60 µm) while allowing solution exchange. Pneumatic valves enable routing of fluids between chambers, and thin-film Au heaters with resistive sensors beneath the selection and amplification chambers provide precise temperature control for thermal elution and PCR cycling.

The workflow of the full-chip SELEX system was similar to a previous description [33] (Figure 1B,C): the SELEX process was performed within the three chambers on a microfluidic chip, with size-exclusion weirs to confine agarose beads in each chamber. The selection chamber is loosely packed with agarose beads with immobilized targets (BAX BH3 in this case), while the amplification chamber is filled with streptavidin-coated beads that have reverse primers on the surface. In later rounds, the counter-selection chamber would have the counter-targets on the agarose beads for the capture of non-specific binders. The flow of liquid was controlled by a syringe pump and pneumatic valves, while heat control was performed by the gold electrode underneath the chambers. Typical operating parameters were as follows: library injection at 10 µL/min, washing at 20 µL/min for 35–45 min (700–900 µL total) depending on round, thermal elution, and on-chip PCR with 12 cycles (95 °C denaturation). A total of nine rounds of microfluidic SELEX were carried out. Each selection round required approximately 3 h, including library incubation, washing, thermal elution, counter-selection, and on-chip amplification. In early rounds, counter-selection was omitted to avoid loss of rare high-affinity binders, while later rounds increased wash duration and reduced library input to raise stringency, reflecting iterative optimization during assay development.

#### Aptamer selection.

A synthetic DNA library synthesized by Integrated DNA Technologies, containing 40 randomized nucleotides (nts) flanked by a 20 nt primer region on both sides, was used as the initial library (5′–TGCAGAAAATAATGCAGGA–N40–GCTGTGATTGTTGGATCAGA–3′). In the first round, 4 nmol (8 µM, 500 µL) of the initial library was used to mix with BAX BH3 peptide (55–74, STKKLSECLKRIGDELDSNM) modified NHS beads. We chose a 40 nt random region as a standard balance of structural diversity, robust PCR, and effective library representation. Longer regions such as 60 nt are more likely to form stable secondary structures, sequester primer sites, increase PCR bias, and yield fewer full-length products during synthesis, so fewer sequences are present and amplifiable [48]. For the BAX BH3 peptide epitope, 40 nt provides sufficient folding capacity without these penalties. The selection buffer (PBSM) contained 137 mM NaCl, 2.7 mM KCl, 2 mM MgCl_2_, 10 mM Na_2_HPO_4_, and 1.8 mM KH_2_PO_4_, adjusted to pH 7.4. This buffer composition was maintained consistently across selection, washing, and elution steps to ensure ionic stability and aptamer folding. The selection partitioning was performed using microfluidic manipulations. During assay development, several optimization steps were introduced to balance enrichment efficiency and stringency. Counter-selection was omitted in the first two rounds to preserve rare high-affinity sequences. Beginning with round 3, counter-selection using non-target protein-conjugated agarose beads was introduced to reduce nonspecific binders. Washing duration was gradually increased from 35 min in early rounds to 45 min in later rounds. In addition, pool input amounts were reduced in later rounds to increase selection pressure. These stepwise adjustments enhanced enrichment while maintaining sequence diversity. After nine rounds of selection, the enriched pools of round 6 and round 9 were sequenced with Illumina 2nd-generation sequencing technologies. Sequences with the highest frequencies were then clustered and synthesized by IDT (Integrated DNA Technologies) for affinity determination. Pierce NHS-Activated Agarose Slurry and streptavidin-coupled agarose beads (Pierce Streptavidin Agarose) were procured from ThermoFisher Pierce (Waltham, MA, USA).

#### Next-generation sequencing sample preparation.

The collected enriched pool samples from SELEX rounds were amplified again on a benchtop thermal cycler (15–20 rounds), and the PCR product was purified with NucleoSpin PCR cleanup Kit (Macherey-Nagel, Bethlehem, PA, USA). The purified dsDNA was then quantified by Spectrophotometer (Implen, Westlake Village, CA, USA). Sample preparation for next-generation sequencing was performed by following KAPA Hyper Prep Kit protocol. First, 250 ng of sample in 50 µL of PCR-grade water was combined with 7 µL of End Repair & A-Tailing Buffer and 3 µL of End Repair & A-Tailing Enzyme Mix. The mixture was gently mixed and incubated at 20 °C for 30 min and at 65 °C for another 30 min. The 60 µL of the resulting product was immediately combined with adapter ligation mix (2.5 µL NextFlex barcode adapter, 7.5 µL PCR-grade water, 30 µL ligation buffer, 10 µL DNA ligase) and mixed gently before brief centrifugation. The ligation reaction mixture was incubated at 20 °C for 20 min. The resulting product was immediately mixed with 0.8× Ampure beads (110 µL product with 88 µL of beads) to perform reaction cleanup. The cleanup suspension was vortexed vigorously before incubating at room temperature (RT) for 15 min. The suspension was then placed atop a magnet to capture beads. After 15 min, the clear supernatant was carefully pipetted out and discarded, and the sediment was washed twice with 200 µL of 80% ethanol while the tube remained on the magnet. Each time, ethanol was carefully removed without disturbing the beads. The remaining beads were dried at RT for 5 min and then removed from the magnet before thoroughly resuspending with 25 µL of elution buffer (5 mM Tris-HCl) and incubated at RT for 2 min to elute DNA off the beads. The tube was placed on a magnet to capture the beads. The collected 22.5 µL of the clear supernatant was combined with 25 µL of KAPA HiFi HotStart ReadyMix (Roche Diagnostics, Indianapolis, IN, USA; #07958927001) and 2.5 µL of primer mix and incubated on a thermal cycler with the following protocol: initial denaturation at 98 °C for 45 s; followed by 6 cycles of 98 °C for 15 s, 60 °C for 30 s, and 72 °C for 30 s; the final extension at 72 °C for 1 min and HOLD on 4 °C. The PCR product was loaded onto a 3% agarose gel (80 mL) for preparatory electrophoresis. The corresponding band at the expected size was cut from the gel and purified with NucleoSpin Gel and PCR Clean-up Kit (Macherey-Nagel; #740611). The purified ligated dsDNA was quantified by the Qubit fluorometer (Thermo Fisher).

#### Sandwich assay.

5′-Amine BAX aptamer candidates were covalently attached to 50 µL of NHS agarose beads before incubating with BAX protein (final concentration 1 µM in PBS) and anti-BAX AF488 (5 µg/mL in PBS) for 1 h. The agarose beads were gently washed with 1× PBS twice and subjected to fluorescence microscopy analysis.

#### Fluorescence polarization (FP) assay.

FP assay for determination of dissociation constant (*K*_D_) values was performed in PBSM buffer in 96-well plates. Briefly, aptamer candidates dissolved in PBSM buffer were conditioned in the following order: 10 min at 95 °C; 10 min on ice; 10 min at RT. The conditioned aptamer candidates (final concentration 200 nM) were then incubated with BAX protein that was also dissolved in PBSM buffer in 96-well plates for 40 min before being analyzed on the plate reader using FP mode.

Agarose beads were used only during selection and characterization and are not included in the biomarker assay, so they do not affect assay sensitivity or specificity.

### 3.2. Colocalization Studies in TK6 Cells

#### Cell line.

TK6 cell line [49] was obtained from Dr. Sally Amundson as a gift and was maintained in a sterile environment with >95% humidity at 37 °C and 5% CO_2_ using culture media containing 500 mL of RPMI 1640 1× medium, with 10% fetal bovine serum, 1% of MEM non-essential amino acids, 1% of sodium pyruvate and 1% of penicillin-streptomycin-glutamine. Cell density was kept below 1 × 10^6^ cells/mL at all times. All experiments were performed using cells within 25 passages.

#### Cell fixation.

Cell fixation was performed similarly to the method previously described [20]. Briefly, TK6 cell samples (<15 mL each) were centrifuged at 200× *g* for 10 min, and the supernatants were discarded. The pellets were resuspended in 250 µL of ice-cold Fixation/Permeabilization solution (from the BD Cytofix/Cytoperm^™^ Fixation/Permeabilization Kit, San Jose, CA, USA; #554714) and incubated at 4 °C for 20 min to allow cell fixation. After the addition of 700 µL of 1× Perm/Wash^™^ Buffer (from the BD Cytofix/Cytoperm^™^ Fixation/Permeabilization Kit, BD Biosciences, San Jose, CA, USA; #554714), the suspensions were centrifuged at 300× *g* for 4 min to remove supernatant. The pellets were then washed twice with 950 µL of 1× Perm/Wash^™^ Buffer, BD Biosciences) before resuspension with 1 mL of 1% (*m*/*v*) bovine serum albumin (BSA) solution in 1× DPBS and preserved at 4 °C.

#### Cell labeling.

Fixed TK6 cells preserved in 1% BSA buffer were first centrifuged at 300× *g* for 4 min, and the supernatant was discarded. The aptamer tagged to Alexa Fluor^®^ 647 (Apt3-AF647, custom purchase from IDT) was diluted in 1× Perm/Wash^™^ Buffer (50 µL) to the final concentration of 500 nM and conditioned in the following order: 10 min at 95 °C; 10 min on ice; 10 min at RT. The pellets were washed with 950 µL of 1× Perm/Wash^™^ Buffer. The cell pellets were then resuspended in 50 µL of the conditioned aptamer (Apt3-AF647) at 500 nM and 50 µL anti-BAX commercial antibody conjugated to Alexa Fluor^®^ 488 (Ab-AF488, BioLegend^®^, San Diego, CA, USA; #633604) dissolved in 1× Perm/Wash^™^ Buffer at 20 µg/mL and incubated for 1 h at RT away from light. After incubation, 900 µL of 1× Perm/Wash™ Buffer was added to the suspension, and the samples were then centrifuged at 300× *g* for 4 min. After discarding the supernatant, the pellets were washed once again with 950 µL of 1× Perm/Wash^™^ Buffer and twice with 950 µL of 1× PBS. If not directly used in the following fluorescence analysis, the pellets were then resuspended in 1 mL of 1× PBS and stored at 4 °C.

#### Imaging flow cytometry.

Samples were concentrated to 50 µL and acquired on the ImageStreamX MkII Imaging Flow Cytometer (Cytek Biosciences, Fremont, CA, USA) at Columbia University Stem Cell Initiative Flow Cytometry core facility with 40× magnification and 488 nm and 642 nm excitation lasers, both set at 150 mW. In each sample, 5000 focused and single cells were acquired based on the gating of gradient root mean square (RMS) feature and area versus aspect ratio features, respectively.

Colocalization analysis was performed on Image Data Exploration and Analysis Software (IDEAS^®^, Luminex ver. 6.2) as follows: Focused cells were selected by visually inspecting captured cell images and using the brightfield (BF) RMS feature. Single cells were gated using a bivariate plot of BF area versus BF aspect ratio, eliminating debris and doublets. Cells that were double-positive for both anti-BAX commercial antibody (Ab- AF488) and aptamer (Apt3 + AF647) were gated using a bivariate plot of signal intensities detected on the 480–560 nm versus 640–745 nm channels. Images with saturated signal were excluded using the raw max pixel feature (the largest pixel intensity) for the 480–560 nm and 640–745 nm channels. Degree of Ab-AF488 and Apt3 + AF647colocalization in doublepositive cells was quantified by the bright detail similarity feature (the log-transformed Pearson’s correlation coefficient of the localized bright spots with a radius of 3 pixels or less within the masked area of the two input images).

### 3.3. Radiation Blood Studies

#### Human ex vivo.

Human blood ex vivo studies were performed as previously described [35]. Briefly, peripheral blood was collected from healthy human donors by venipuncture (written consent obtained, approved by Columbia University IRB AAAS7621). Mock and 3 Gy irradiation of the human whole blood samples were performed on X-RAD 320 Biological Irradiator (Precision X-Ray Inc., North Brandford, CT, USA). A custom filter (1.5 mm + Al 0.25 mm Cu + 1.25 mm Sn), 320 mV, 12.5 mA, and a 40 cm focus to skin distance (FSD) were used to achieve 1 Gy/min irradiation rate. Blood aliquots (100 µL) were cultured in 900 µL RPMI media (with 15% FBS and 1% Pen-Strep) at 95 °C, 5% CO_2_ for 24 h. Samples were then treated with RBC lysis buffer (Invitrogen; Carlsbad, CA, USA; #00–4333-57) fixed, and permeabilized (Cytofix/Cytoperm^™^ Fixation/Permeabilization Solution Kit, BD Biosciences, #554714). Samples were then stained with pre-conditioned (95 °C 5 min, followed by incubation on ice 5 min) 0.5 µM Apt3 for 1 h in the dark at RT. After very gently washing twice with PBS, samples were stored in 1 mL PBS until sample acquisition.

#### Mouse in vivo.

All animal experiments were approved by Columbia University Institutional Animal Care and Use Committee (IACUC; approved protocol #AABA9506, approval date 23 June 2021) and were conducted under all relevant federal and state guidelines. These methods were performed in accordance with ARRIVE guidelines (https://arriveguidelines.org, accessed on 7 October 2025). Male C57BL/6 mice, aged 12 weeks, were purchased from Charles River Laboratory and housed (five animals maximum/ventilated cage) in the animal facilities at the Columbia University Irving Medical Center (CUIMC) under specific pathogen-free conditions and maintained at a temperature of 20 ± 3 °C and humidity of 40–60% (with a 12 h light/dark cycle). Mouse diet comprised 5053 Irradiated PicoLab^®^ Rodent Diet 20 (Lab Diet^®^, Arden Hills, MN, USA) and water ad libitum. Mouse in vivo studies were performed as previously described [20]. Briefly, mice (aged 15–16 weeks; *n* = 2) were randomly selected and mock or 2.5 Gy X-irradiated using an X-RAD biological irradiator (1.5 mm Al + 0.25 mm Cu + 0.75 mm Sn filter, FSD 48 cm, 320 kVp, 12.5 mA at 1 Gy/min dose rate and returned to a clean cage with wet food pellets and hydrogel packs (Clear H_2_O, Westbrook, ME, USA) placed on the cage floor. Mice were euthanized at 24 h post-irradiation by CO_2_ inhalation, followed by cervical dislocation in accordance with CUIMC-IACUC guidelines. Peripheral blood was collected by cardiac puncture.

Whole blood sample aliquots (100 µL) were prepared for lysis, fixation, and perme-abilization as described above for the human ex vivo blood samples. Samples were then stained with pre-conditioned (95 °C for 5 min, followed by incubation on ice for 5 min) 0.33 µM Apt3 for 1 h in the dark at RT. After very gently washing twice with PBS, samples were stored in 1 ml PBS until sample acquisition.

#### Imaging flow cytometry.

Samples were concentrated to 50 µL and acquired on ImageStreamX MkII at Columbia University Center for Radiological Research with 40× objective and 488 nm excitation laser set to 35 mW and 200 mW for human and mouse samples, respectively. Similarly to the method previously described [20], a uniform analysis template on IDEAS^®^, Luminex ver. 6.2 was used to measure MFI detected on the 480–560 nm channel in focused, single, healthy leukocytes. Unstained samples were examined to account for increases in fluorescence intensity due to autofluorescence.

## 4. Conclusions

In summary, this study established a rapid microfluidic SELEX method for isolating aptamers targeting BAX protein as a potential biomarker for radiological triage. By employing a 20-amino acid BH3 peptide segment instead of the full-length protein, we improved the efficiency of the selection process and obtained three aptamer candidates (Apt1–3). Comparative analyses identified Apt3 as the most promising candidate, while Apt1 and Apt2 showed limited or inconclusive performance in bead-based and fluorescence assays. Apt3 was further validated through colocalization with a commercial anti-BAX antibody and demonstrated the ability to detect radiation-induced BAX expression in both ex vivo human blood and in vivo mouse blood assays. These data suggest that novel Apt3 is capable of binding BAX protein in a cellular context, indicating its potential use for intracellular BAX measurements after radiation exposure. Although the aptamer signal showed colocalization with BAX immunostaining consistent with the known subcellular dynamics of BAX, cross-reactivity testing with related proteins and structurally similar molecules will be required to rigorously confirm the specificity of Apt3. Future studies will be necessary to use larger human and mouse blood sample sizes to determine the dose range and linearity of aptamer-based intracellular BAX detection after irradiation. Demographic studies will be important to rigorously assess inter-individual variability, improve statistical power, and validate the robustness and generalizability of this aptamer-based approach for detecting intracellular BAX protein. Given the central role of BAX in apoptosis, the cross-reactivity of the human aptamer in mouse lymphocytes underscores the conserved nature of BAX and supports its broader applicability. Overall, this proof-of-concept highlights the feasibility of microfluidic SELEX for intracellular aptamer discovery and demonstrates its potential for biodosimetry and related translational applications.

## Supplementary Material

supplementary material

The following supporting information can be downloaded at: https://www.mdpi.com/article/10.3390/radiation5040030/s1, Figure S1. Antibody detection of intracellular BAX protein in mouse peripheral blood. C57BL/6 mice were X-irradiated with 0 and 2.5 Gy (one mouse at each dose) and sacrificed after 24 h. Peripheral blood leukocytes from each mouse were stained with commercial anti-BAX antibody, and MFI for each dose was measured by IFC. Fold changes of dose-dependent BAX expression were calculated as 2.5 MFI/0 Gy MFI. Figure S2. Fluorescent microscopy image of SELEX pool characterization. Three SELEX attempts started from different aliquots of the same ssDNA library with 30 N random region were performed on BAX BH3 peptide SELEX and named BAX#2, BAX#3, and BAX#4. The eluents collected from the last round of SELEX on the microfluidic chip for each attempt were then amplified with fluorescent primers and conditioned to yield the fluorescent ssDNA. The ssDNA was then incubated with BAX BH3 immobilized on beads, and then subjected to fluorescent microscopy. The red rectangle highlights the attempt that was subject to NGS in the main text.

## Figures and Tables

**Table 1.** Top nine clusters in BAX BH3 peptide microfluidic SELEX NGS. Primer regions are hidden. Unique sequences are color-coded and termed. Blue: Apt1, Red: Apt2, Green: Apt3.

Round	#	Sequence	Cluster Size
	1	GCAGGGGGGTGGGTGGGTGGATGGTACTGCGTGTTGTGGC	110,644
	2	GGGTGGGATTGGGGGACCGGTGTATTTGGGGGTAGGCTTG	43,606
	3	GCAGGGGGGTGGGTGGGTGGATGGTACTGCGTGTTGTGTG	6043
	4	CATTGTGGCGGGTAGGTTGGAGGGTGGTTGGGGCTTATGG	3341
6	5	GCAGGGGGGTGGGTGGGTGGATGGTACTGCGTGTTGTGAC	1168
	6	GCAGGGGGGTGGGCGGGTGGATGGTACTGCGTGTTGTGGC	1115
	7	GCAGGGGGGTGGGTGGGTGGATGGTACTGCGTGTTGTGCC	1038
	8	GCAGGGGGGTGGGTGGGTGGATGGTACTGCGTGTTGCGGC	858
	9	GCAGGGGGGTGGGTGGGTGGATGGTGCTGCGTGTTGTGGC	829
	1	CATTGTGGCGGGTAGGTTGGAGGGTGGTTGGGGCTTATGG	165,995
	2	GGGTGGGATTGGGGGACCGGTGTATTTGGGGGTAGGCTTG	104,653
	3	CACTGTGGCGGGTAGGTTGGAGGGTGGTTGGGGCTTATGG	6984
	4	CATTATGGCGGGTAGGTTGGAGGGTGGTTGGGGCTTATGG	2200
9	5	GCAGGGGGGTGGGTGGGTGGATGGTACTGCGTGTTGTGGC	1736
	6	CATTGTGGCGGGTAGGTTGGAGGGTGGCTGGGGCTTATGG	1209
	7	GGGTGGGATTGGGGGACCGGTGTATCTGGGGGTAGGCTTG	1110
	8	CATTGTGGCGGGTAGGTTGGAGGGTGGTTGGGGCTTGTGG	737
	9	CATTGTGGCGGGTAGGTTGGAGGGTGGTTGGGGCTTATTG	465

## Data Availability

All experimental data supporting the findings of this study are contained within the article and its Supplementary Materials. No additional data are available.
