# Supplementary material for: Microfluidic Isolation of Aptamers for Intracellular Measurement of Radio-Responsive Proteins
Source: Radiation (Basel). Author manuscript; Available in PMC 2026 Jul 16. (PMC13372225; doi:10.3390/radiation5040030)
Supplement: supplementary material [file NIHMS2162745-supplement-supplementary_material.pdf]

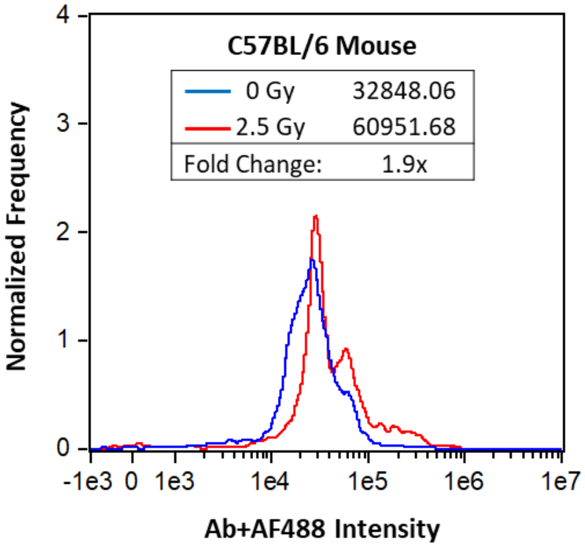

Figure S1. Antibody detection of intracellular BAX protein in mouse peripheral blood. C57BL/6 mice were X-irradiated with 0 and 2.5 Gy (1 mouse at each dose) and sacrificed after 24 hours. Peripheral blood leukocytes from each mouse was stained with commercial anti-BAX antibody, and MFI for each dose was measured by IFC. Fold changes of dose-dependent BAX expression were calculated as 2.5 MFI / 0 Gy MFI.

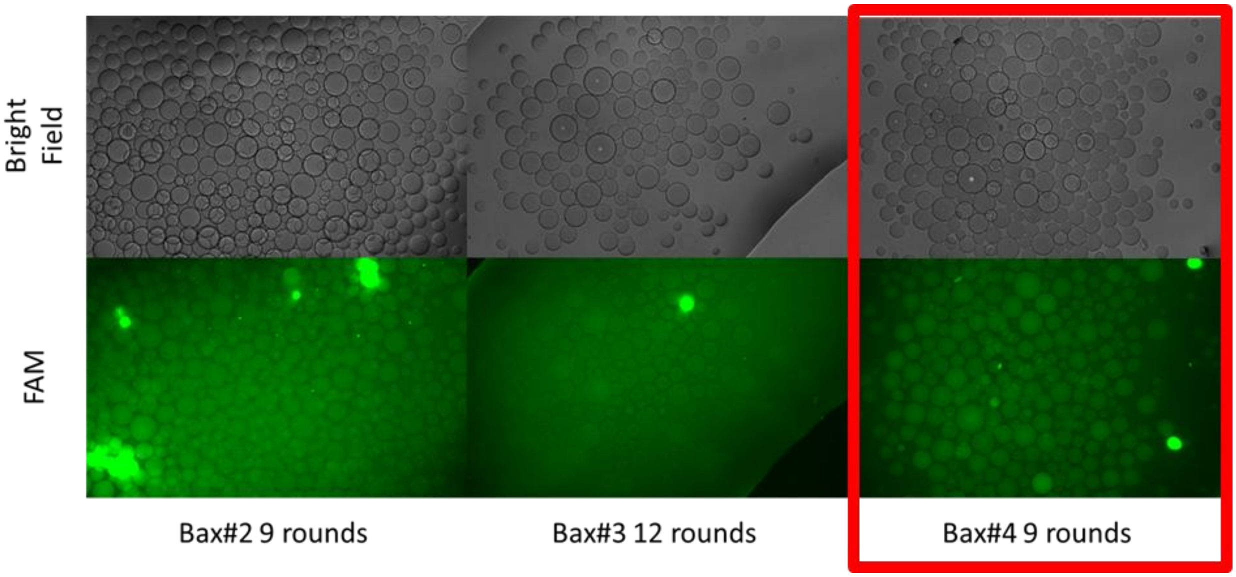

Figure S2. Fluorescent microscopy image of SELEX pool characterization. Three SELEX attempts started from different aliquots of the same ssDNA library with 30 N random region were performed on BAX BH3 peptide SELEX and named BAX#2, BAX#3, and BAX#4. The eluents collected from the last round of SELEX on the microfluidic chip for each attempt were then amplified with fluorescent primers and conditioned to yield the fluorescent ssDNA. The ssDNA was then incubated with BAX BH3 immobilized on beads and then subjected to fluorescent microscopy. The red rectangular highlights the attempt that was subject to NGS in the main text.
